# Paradigm shift in inflammatory bowel disease management—advanced therapy utilization, persistence, and outcomes in a United Arab Emirates cohort during 2015-2025

**DOI:** 10.1093/crocol/otag057

**Published:** 2026-06-16

**Authors:** Hosameldin Abdelrahman Ahmed, Mohamed Nasir Alzaabi, Thaer Khaleel Swaid, Shaima Wasim Khan, Maitha Al Hosani, Noorah Al Hosani, Maryam A Alahmad, Nadeen Mamon Omar, Enas Fouad Ahmed, Ishtiaq Ahmed, Kishore Kumar Chitra Kumar, Laurette L Bukasa, Tine Jess, Mohammed Nabil Quraishi

**Affiliations:** Department of Gastroenterology, Sheikh Shakhbout Medical City, Abu Dhabi, United Arab Emirates; Department of Gastroenterology, Sheikh Shakhbout Medical City, Abu Dhabi, United Arab Emirates; Department of Gastroenterology, Sheikh Shakhbout Medical City, Abu Dhabi, United Arab Emirates; Department of Gastroenterology, Sheikh Shakhbout Medical City, Abu Dhabi, United Arab Emirates; Department of Gastroenterology, Sheikh Shakhbout Medical City, Abu Dhabi, United Arab Emirates; Department of Gastroenterology, Sheikh Shakhbout Medical City, Abu Dhabi, United Arab Emirates; Department of Gastroenterology, Sheikh Shakhbout Medical City, Abu Dhabi, United Arab Emirates; Department of Gastroenterology, Sheikh Shakhbout Medical City, Abu Dhabi, United Arab Emirates; Department of Gastroenterology, Sheikh Shakhbout Medical City, Abu Dhabi, United Arab Emirates; Department of Gastroenterology, Sheikh Shakhbout Medical City, Abu Dhabi, United Arab Emirates; Department of Gastroenterology, Sheikh Shakhbout Medical City, Abu Dhabi, United Arab Emirates; Abu Dhabi Health Data Services, Abu Dhabi, United Arab Emirates; Great Ormond Street Institute for Child Health, UCL, London, United Kingdom; Center for Molecular Prediction of Inflammatory Bowel Disease, Department of Clinical Medicine, Aalborg University, Copenhagen, Denmark; Department of Gastroenterology and Hepatology, Aalborg University Hospital, Aalborg, Denmark; Department of Gastroenterology, Sheikh Shakhbout Medical City, Abu Dhabi, United Arab Emirates; Institute of Cancer and Genomic Sciences, University of Birmingham, Birmingham, United Kingdom; College of Medicine and Health Sciences, Khalifa University of Science and Technology, Abu Dhabi, United Arab Emirates

**Keywords:** inflammatory bowel disease, advanced therapy, treatment persistence, anti-IL-23, real-world evidence

## Abstract

**Background:**

The therapeutic landscape for inflammatory bowel disease (IBD) has expanded rapidly beyond anti-tumor necrosis factor (anti-TNF) agents. We characterized the evolution of advanced therapy utilization, persistence, and outcomes in a United Arab Emirates (UAE) cohort during 2015–2025.

**Methods:**

This prospective registry study from a tertiary IBD facility in the UAE included 508 patients (333 Crohn’s disease [CD], 175 ulcerative colitis [UC]). Advanced therapies were grouped by mechanism of action. We compared prescribing patterns between “Pre-2023” and “2023 and Beyond” eras. Treatment persistence was analyzed using Kaplan-Meier methods and Cox proportional hazards models.

**Results:**

Of 508 patients, 380 (74.8%) received advanced therapy. In CD, first-line anti-TNF use fell from 76% (pre-2023) to 42% (2023+), with anti-IL-23 agents emerging as a major first-line option (32%). Median time to first advanced therapy decreased dramatically: CD from 23.0 to 2.0 months (*P* < .0001) and UC from 37.0 to 3.0 months (*P* < .0001). Anti-IL-23 agents demonstrated significantly superior persistence in CD first-line therapy (*P* = .011 vs. anti-TNF). In multivariable analysis, anti-IL-23 therapy was associated with significantly lower discontinuation risk (HR: 0.35, 95% CI: 0.19-0.64, *P* = .0008). Infliximab persistence appeared to improve over time, with significantly better 1-year persistence in 2024+ versus 2018–2019 (pairwise *P* = .020; overall trend *P* = .05). A shift in UC second-line prescribing was also observed (*P* = .048). Early surgery rates were low across both eras (CD: 6.5% vs. 3.9%; UC: 2.5% vs. 1.9%) with no significant difference in 35-month surgery-free survival, though this analysis was underpowered.

**Conclusions:**

IBD management in the UAE has undergone a rapid paradigm shift since 2023, characterized by earlier therapy initiation and diversification away from anti-TNF dominance, particularly in CD. Superior persistence of anti-IL-23 agents in our cohort is consistent with this evolution in clinical practice and supports their further evaluation.

## Graphical Abstract

**Figure:**
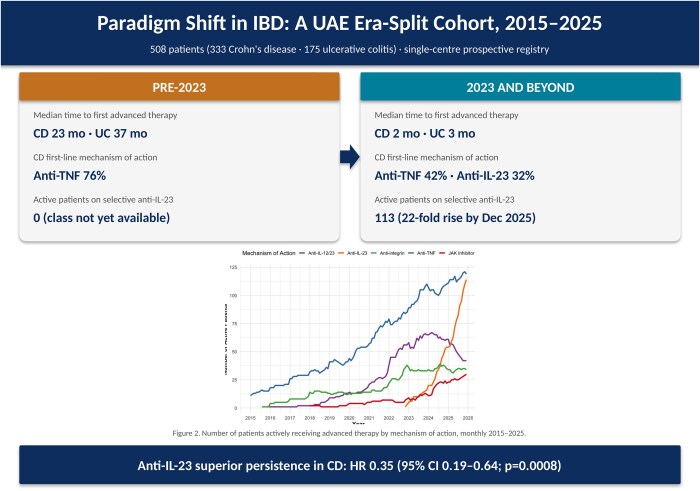

## Introduction

The management of inflammatory bowel disease (IBD), encompassing Crohn’s disease (CD) and ulcerative colitis (UC), has been revolutionized by the advent of advanced therapies. The introduction of anti-tumor necrosis factor (anti-TNF) agents marked a paradigm shift from conventional immunosuppressants to targeted biologic therapy, significantly altering the natural history of the disease.^1^ In recent years, the therapeutic armamentarium has expanded at an unprecedented rate with the approval of agents targeting novel pathways, including anti-integrins, anti-interleukin (IL)-12/23, selective anti-IL-23 agents, and Janus kinase (JAK) inhibitors.^2^

This rapid expansion presents a paradigm shift for clinicians. The traditional anti-TNF-first approach is now challenged by a multi-mechanism of action (MOA) landscape, raising critical questions about optimal first-line therapy positioning and subsequent sequencing. This is particularly relevant in the context of the Middle East, a region with a rapidly rising IBD incidence.^3^^,^^4^ Recent epidemiological analysis from our center has highlighted the accelerating clinical burden of IBD in the United Arab Emirates (UAE), with a growing proportion of patients presenting with complicated disease phenotypes.^5^ This increasing disease burden necessitates a parallel evolution in management strategies to ensure that the most effective therapies are utilized to optimize patient outcomes.^6–8^

While head-to-head trials have provided some guidance, real-world evidence is crucial for understanding how clinicians adapt their strategies as new data and options become available.^9^^,^^10^ The UAE’s unique healthcare setting provides a valuable opportunity to observe this therapeutic evolution; its local population is covered by comprehensive national health insurance, which underpins a system with rapid drug approval processes, early access to therapies following international registration, and a notable lack of the stringent positioning restrictions seen in other parts of the world. The therapeutic revolution may have a distinct pattern around years 2022–2023, moving from the anti-TNF-dominant era to a modern, multi-MOA era^11^ following the regulatory approval and clinical availability of the first selective anti-IL-23 inhibitors and selective JAK-1 inhibitors for IBD indications in our region. Therefore, as a continuation of our work exploring this epidemiological shift, the primary aim of this analysis was to characterize how clinical practice has responded to both the rising disease burden and the availability of novel therapies. We sought to define the evolution of advanced therapy utilization, persistence, sequencing, and associated surgical outcomes in our IBD cohort during 2015–2025. To facilitate this, we defined two distinct therapeutic eras: a “Pre-2023” period dominated by established therapies and a “2023 and Beyond” period marked by the introduction of novel agents to understand how clinical practice has responded to both the rising disease burden and the expanding therapeutic armamentarium.

## Methods

### Study design, population, and data source

The study was based on the prospectively maintained UAE Epi-IBD registry at Sheikh Shakhbout Medical City (SSMC), Abu Dhabi, a large tertiary referral center in the UAE. As previously described,^5^ this study included only patients under active follow-up at our IBD center at the analysis censoring date (December 28, 2025) who had a comprehensive and accurately documented longitudinal treatment history. The cohort included all adult patients (≥18 years) with a confirmed diagnosis of CD or UC based on standard endoscopic, histological, and radiological criteria. Patients who had relocated, transferred care to another provider, or were otherwise lost to follow-up were not included in the analytic cohort. For the present study, data were extracted from the electronic registry, and follow-up for all patients was censored on December 28, 2025. We included baseline demographic information, disease-specific characteristics (Montreal classification for CD, disease extent for UC), and a complete history of advanced therapy use, including initiation and discontinuation dates for each line of therapy. For analysis, advanced therapies were grouped by MOA: anti-TNF, anti-integrin, anti-IL-12/23, anti-IL-23, and JAK inhibitors. Surgical data included the date of the first major IBD-related abdominal surgery.

### Drug approval and availability in the UAE

UAE registration dates for the advanced therapy classes used in this cohort were verified against the Emirates Drug Establishment registered medications list. Anti-TNF agents have been UAE-registered since 2000 (first as infliximab, with adalimumab and the remaining anti-TNF agents registered subsequently), providing continuous anti-TNF availability throughout the study period. Anti-integrin therapy (first registered as vedolizumab) became available in 2015, and anti-IL-12/23 therapy (ustekinumab) in 2014; both classes preceded the start of our cohort. The JAK inhibitor class was first UAE-registered in 2013 (as tofacitinib), with upadacitinib arriving subsequently in November 2021. The selective anti-IL-23 class entered the UAE shortly afterward (first registered as risankizumab in its 360 mg/2.4 mL IBD formulation in August 2022), with subsequent anti-IL-23 agents (mirikizumab, guselkumab) registered later in the study period. Sphingosine-1-phosphate receptor modulators are not registered for UC in the UAE.

### Outcomes and definitions

Primary outcomes were: (1) the evolution of advanced therapy utilization patterns by MOA across the pre-2023 versus 2023-and-beyond eras; and (2) treatment persistence on advanced therapies overall and by MOA. Secondary outcomes were: (1) time from diagnosis to first advanced therapy by era; (2) treatment sequencing pathways by era; (3) 35-month surgery-free survival by era; (4) predictors of first IBD-related abdominal surgery; and (5) the relationship between Montreal disease behavior at diagnosis and total advanced therapy exposure. Operational definitions: “Treatment discontinuation” was defined as the physician-documented stop date in the registry, encompassing primary non-response, secondary loss of response, adverse event, patient choice, planned pregnancy, or surgical intervention. “First IBD-related abdominal surgery” was defined as the first major IBD-related bowel resection, colectomy, ileostomy or colostomy formation, or other intra-abdominal IBD surgery, recorded in the registry; perianal abscess drainage and seton placement were excluded. “Time to first advanced therapy” was defined as the interval from confirmed diagnosis to the date of first dose of any advanced therapy (anti-TNF, anti-integrin, anti-IL-12/23, anti-IL-23 or JAK inhibitor); patients diagnosed prior to referral to the center were anchored to their original confirmed diagnosis date as recorded in source documentation. “Active therapy use” on a given calendar month was defined as a documented start date on or before, and stop date on or after, that month.

### Statistical analysis

All statistical analyses were performed using R statistical software (Version 4.3.2).^12^ Descriptive statistics were used to summarize baseline cohort characteristics; continuous variables were reported as medians and interquartile ranges (IQR) and compared using the Wilcoxon rank-sum test, while categorical variables were reported as counts and percentages and compared using the Chi-squared or Fisher’s exact test. A two-sided *P-*value of ≤.05 was considered statistically significant. Pairwise log-rank comparisons were adjusted for multiplicity using the Benjamini-Hochberg method.

The prevalence of active therapy use over time was determined by calculating the number of unique patients on each MOA on a monthly basis from January 2015. To analyze the evolution of prescribing patterns, new therapy initiations were categorized into two eras: a “Pre-2023” era (January 1, 2015–December 31, 2022) and a “2023 and Beyond” era (January 1, 2023–December 28, 2025). The 1 January 2023 boundary was selected a priori because it corresponds to the regulatory approval and clinical availability of the first selective anti-IL-23 inhibitor (risankizumab) and the first selective JAK-1 inhibitor (upadacitinib) for IBD indications in the UAE, marking the transition from a landscape dominated by anti-TNF, anti-integrin and anti-IL-12/23 agents to one with multiple alternative mechanisms. The proportional use of each MOA across lines of therapy was compared between eras using the Chi-squared test. Treatment pathways were visualized using Sankey diagrams, stratified by disease and initiation era. For the analysis of time to first advanced therapy initiation, only patients diagnosed up to 2024 were included to ensure a minimum of 12 months follow-up.

Treatment persistence was analyzed using the Kaplan-Meier method, with treatment discontinuation as the primary event. Patients remaining on therapy were censored at the study end date. Differences in persistence curves between MOAs were assessed using the log-rank test, with pairwise comparisons adjusted using the Benjamini-Hochberg method. A focused analysis of 1-year infliximab persistence grouped initiations into biennial eras (2018–2019, 2020–2021, 2022–2023, and 2024–2025) to assess for temporal improvements. To identify predictors of treatment discontinuation, univariable and multivariable Cox proportional hazards models were constructed. Variables were selected a priori on the basis of clinical relevance and prior published literature on persistence predictors. The multivariable model for the entire advanced-therapy-exposed cohort included drug class (reference: anti-TNF), IBD subtype (in the pooled model only), line of therapy, age at diagnosis, smoking history (current/ex/never; reference: never), and disease duration at therapy initiation. The CD-only multivariable model additionally included Montreal disease behavior (reference: B1), disease location (reference: ileo-colonic), and perianal disease (reference: no). The UC-only multivariable model additionally included disease extent (reference: proctitis). Proportional hazards assumptions were assessed by Schoenfeld residuals. These analyses were performed for the entire cohort and for CD and UC subgroups.

The primary surgical outcome was the time from IBD diagnosis to the first major IBD-related abdominal surgery. Surgery-free survival was analyzed using the Kaplan-Meier method. For this specific analysis, follow-up was censored at 35 months to allow for a standardized comparison of early surgical risk, mitigating bias from the shorter maximum follow-up time available in the second era (January 2023 to December 2025). Predictors of requiring surgery were identified using a multivariate logistic regression model, with variables selected a priori based on clinical knowledge and established literature. The association between Montreal phenotype and the total number of advanced therapies required was assessed using the Kruskal-Wallis test.

### Ethical considerations

The study was approved by the Institutional Review Board at Sheikh Shakhbout Medical City – (SSMCREC-585; date approved March 2, 2025). All data used in this analysis were anonymized to protect patient confidentiality. Informed consent was waived by the board due to the retrospective nature of the study. The study was conducted in compliance with the Declaration of Helsinki.

## Results

### Cohort demographics

The analysis included 508 patients from the IBD cohort (333 CD, 175 UC), the majority of whom were of Emirati nationality (80%). Baseline demographic and clinical characteristics are detailed in Table 1. Patients with CD were younger at diagnosis (median 24 vs. 28 years, *P* < .001) and the cohort had a higher proportion of males (64% vs. 50%, *P* = .003) than patients with UC. A history of smoking was also more prevalent among CD patients (18.5% vs. 4.5%, *P* < .001). Of the 508 patients, 380 (74.8%) had been exposed to at least one advanced therapy. For their first-line treatment, anti-TNF therapy was the most frequently initiated MOA (*n* = 223). However, other MOAs, including anti-integrin (*n* = 46), anti-IL-12/23 agents (*n* = 60), anti-IL-23 agents (*n* = 41), and JAK inhibitors (*n* = 10), were also commonly used. Of the patients who initiated a first-line therapy, 177 (46.6%) went on to receive a second or subsequent line of therapy. For a small subset of the most refractory patients (*n* = 9), combination advanced therapy (the use of two concurrent biologics and/or small molecules) was required for refractory disease, rather than for overlapping immune-mediated inflammatory diseases. This cohort consisted predominantly of patients with CD (7/9, 78%) and included various novel pairings such as anti-TNF with anti-integrin, and anti-IL-23 agents with either anti-TNF therapy or JAK inhibitors.

**Table 1:** Baseline demographic and clinical characteristics of the IBD cohort 2015-2025.

	Overall *N* = 508^a^	Crohn’s disease *N* = 333^a^	Ulcerative colitis *N* = 175^a^	*P* value^b^
**Age (years)**	33 (25, 42)	32 (23, 41)	36 (26, 44)	.011
**Gender**				.003
** Female**	207 (41%)	120 (36%)	87 (50%)	
** Male**	301 (59%)	213 (64%)	88 (50%)	
**Nationality**				.024
** Caucasian**	6 (1.2%)	4 (1.2%)	2 (1.1%)	
** Emirati**	407 (80%)	273 (82%)	134 (77%)	
** Non-Emirati Arab**	70 (14%)	47 (14%)	23 (13%)	
** Other**	2 (0.4%)	1 (0.3%)	1 (0.6%)	
** South Asian**	23 (4.5%)	8 (2.4%)	15 (8.6%)	
**Smoking history**				<.001
** Current smoker**	51 (10%)	45 (14%)	6 (3.4%)	
** Ex-smoker**	16 (3.1%)	14 (4.2%)	2 (1.1%)	
** Non smoker**	438 (86%)	273 (82%)	165 (94%)	
** Unspecified**	3 (0.6%)	1 (0.3%)	2 (1.1%)	
**Age at diagnosis (years)**	25 (19, 34)	24 (18, 33)	28 (22, 36)	<.001
**Advanced therapy naive**	128 (25%)	59 (18%)	69 (39%)	<.001
**Number of advanced therapies**				<.001
** 0**	128 (25%)	59 (18%)	69 (39%)	
** 1**	203 (40%)	140 (42%)	63 (36%)	
** 2**	94 (19%)	70 (21%)	24 (14%)	
** 3**	41 (8.1%)	31 (9.3%)	10 (5.7%)	
** 4**	22 (4.3%)	16 (4.8%)	6 (3.4%)	
** 5 or more**	20 (3.9%)	17 (5.1%)	3 (1.7%)	
**Ever exposed to infliximab**	214 (42%)	161 (48%)	53 (30%)	<.001
**Ever exposed to adalimumab**	105 (21%)	92 (28%)	13 (7.4%)	<.001
**Ever exposed to golimumab**	2 (0.4%)	1 (0.3%)	1 (0.6%)	>.9
**Ever exposed to certolizumab**	2 (0.4%)	2 (0.6%)	0 (0%)	.5
**Ever exposed to vedolizumab**	86 (17%)	38 (11%)	48 (27%)	<.001
**Ever exposed to ustekinumab**	124 (24%)	97 (29%)	27 (15%)	<.001
**Ever exposed to risankizumab**	68 (13%)	66 (20%)	2 (1.1%)	<.001
**Ever exposed to guselkumab**	56 (11%)	46 (14%)	10 (5.7%)	.006
**Ever exposed to mirikizumab**	2 (0.4%)	0 (0%)	2 (1.1%)	.12
**Ever exposed to tofacitinib**	9 (1.8%)	1 (0.3%)	8 (4.6%)	.001
**Ever exposed to upadacitinib**	41 (8.1%)	23 (6.9%)	18 (10%)	.2
**Ever on advanced combination therapy**	9 (1.8%)	7 (2.1%)	2 (1.1%)	.7

a Median (Q1, Q3); *n*/*N* (%); *n* (%).

b Statistical comparison between the Crohn’s disease and ulcerative colitis cohorts—Wilcoxon rank sum test; Pearson’s chi-squared test; Fisher’s exact test.

### Time to first advanced therapy

**Figure 1:**
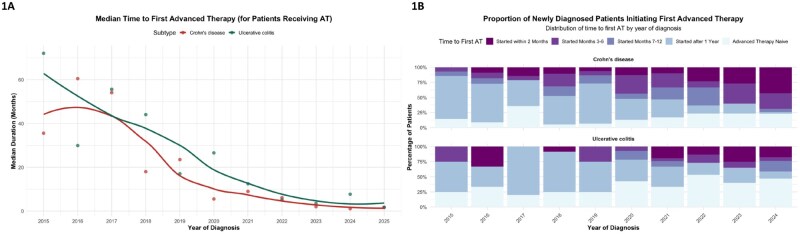
Time to first advanced therapy (ATx) initiation from diagnosis. (A) For patients with CD, the median time from diagnosis to first ATx fell significantly from 23.0 months in the pre-2023 era to just 2.0 months from 2023 onward (*P* < .0001). A significant reduction was also observed for UC, from 37.0 to 3.0 months (*P* < .0001). (B) The proportion of all newly diagnosed patients initiating ATx is shown by year of diagnosis (2015-2024; patients diagnosed in 2025 were excluded to ensure minimum 12 months of follow-up).

During 2015–2024, contrasting trends in time to first advanced therapies were observed between CD and UC; only patients diagnosed up to 2024 were included to ensure a minimum of 12 months of follow-up for assessment of early therapy initiation (Figure 1A and B). The proportion of patients with UC not commencing advanced therapy within the first year fluctuated, stabilizing in the 2021–2024 period, with 47.1% of newly diagnosed UC patients in 2024 remaining advanced therapy naïve. This was in stark contrast to CD, which showed a clear temporal trend toward earlier and more frequent use of advanced therapies. The proportion of CD patients commencing therapy within the first year progressively increased from 14.3% in 2015 to 77.1% in 2024, with a corresponding decrease in those remaining advanced therapy naïve at 12 months (from 71.4% in 2015 to 22.9% in 2024).

Among those patients who received an advanced therapy, the median time from diagnosis to its commencement was significantly shorter in the 2023-and-beyond era. For patients with CD, this time fell from 23.0 months in the pre-2023 era to just 2.0 months in the subsequent period (*P* < .0001). A significant reduction was also observed for UC, falling from 37.0 months to 3.0 months (*P* < .0001).

### Overall trends in advanced therapy utilization

**Figure 2:**
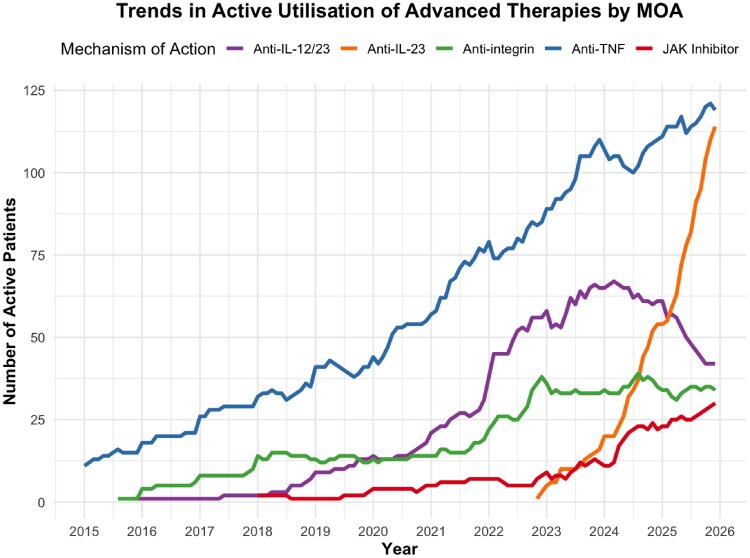
Trends in active utilization of advanced therapies by mechanism of action (MOA). The line graph displays the total number of patients actively receiving advanced therapies each month from 2015 to 2025, grouped by MOA.

The monthly prevalence of patients actively receiving advanced therapies, stratified by MOA, is displayed in Figure 2. In January 2015, 44 unique patients were actively receiving advanced therapy, all on anti-TNF agents. By January 2020, the active cohort had expanded to 89 patients (anti-TNF *n* = 60, anti-integrin *n* = 13, anti-IL-12/23 *n* = 14, JAK inhibitor *n* = 4). By January 2023, immediately before the regulatory availability of selective anti-IL-23 and selective JAK-1 inhibitors for IBD in our region, 200 unique patients were actively on advanced therapy (anti-TNF *n* = 99, anti-integrin *n* = 36, anti-IL-12/23 *n* = 58, anti-IL-23 *n* = 5, JAK inhibitor *n* = 9). By the censoring date of 28 December 2025, this had risen to 337 active patients (anti-TNF *n* = 128, anti-integrin *n* = 34, anti-IL-12/23 *n* = 41, anti-IL-23 *n* = 113, JAK inhibitor *n* = 29). The most striking single change was a 22-fold increase in patients actively on selective anti-IL-23 inhibitors between January 2023 and December 2025 (5 to 113), with anti-IL-23 emerging as the second most prevalent class by the end of the study period. Anti-TNF prevalence continued to grow over the same window but at a markedly attenuated rate, while the number of patients on anti-IL-12/23 declined as switches to selective anti-IL-23 agents accumulated.

### Evolution of prescribing patterns: pre-2023 vs 2023 and beyond

**Figure 3:**
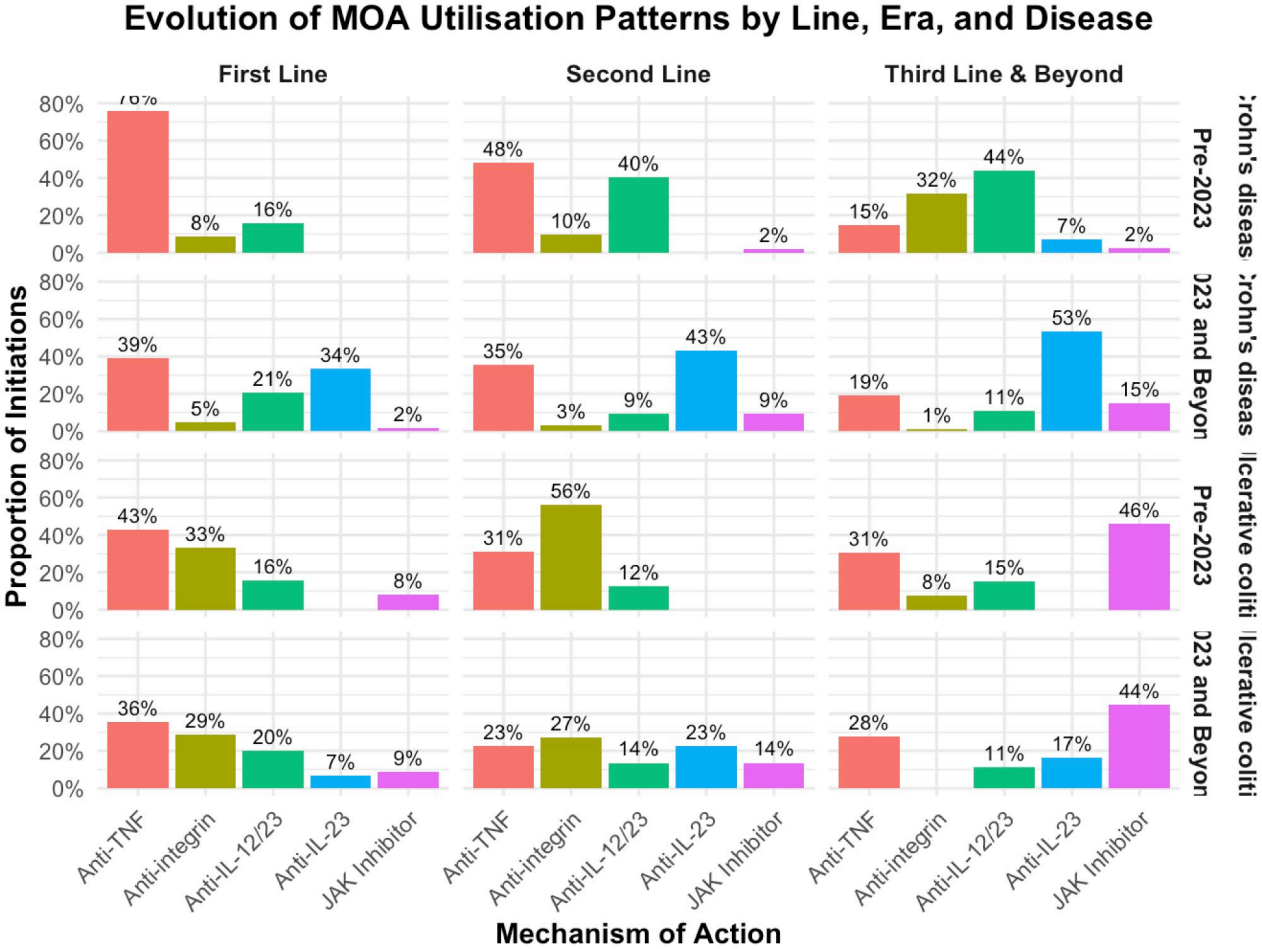
Evolution of mechanism of action (MOA) utilization patterns by line of therapy, era, and disease. The faceted bar charts show the proportional use of each MOA for new therapy initiations.

To further investigate the shift in prescribing, we compared the proportional use of different MOAs for new therapy initiations before 2023 versus 2023 and beyond, stratified by disease and line of therapy (Figure 3). For CD, a statistically significant shift in prescribing patterns was observed across all lines of therapy (*P* < .001 for first, second, and third line & beyond). Before 2023, first-line therapy was dominated by anti-TNF agents (*n* = 94, 76.4% of initiations), which fell to 41.5% (*n* = 49) in 2023 and beyond. This was accompanied by the emergence of anti-IL-23 agents as a major first-line option (*n* = 38, 32.2% of initiations in 2023 and beyond). A similar trend was seen in the second line, where the proportion of anti-TNF initiations decreased (from *n* = 25, 47.2% to *n* = 24, 35.3%) and anti-IL-23 agents became the most common choice (*n* = 29, 42.6%). The most dramatic shift was in third-line and beyond, where anti-IL-23 agents, reflecting their recent introduction into clinical practice, grew from just *n* = 3, 7.3% of initiations before 2023 to account for *n* = 42, 51.9% in 2023 and beyond, supplanting anti-integrin and anti-IL-12/23 agents. In a small group of refractory patients (*n* = 9), dual advanced therapy, using two biologics and/or small molecules at once, was used for treatment-resistant disease. Most had CD (7/9, 78%) and predominantly received an anti-IL-23 agent paired with either anti-TNF or a JAK inhibitor. In contrast, for UC, prescribing patterns showed variable shifts over time. While first-line prescribing remained stable (*P* = .42), a statistically significant shift in second-line prescribing patterns emerged (*P* = .048), with increased use of anti-IL-23 and JAK inhibitors. Anti-TNF (*n* = 23, 44.2% pre-2023; *n* = 18, 37.5% 2023 and beyond) and anti-integrin agents (*n* = 17, 32.7% pre-2023; *n* = 13, 27.1% 2023 and beyond) remained the most common choices for first-line therapy during the period under study. For third-line and beyond, no statistically significant difference was observed (*P* = .30).

### Treatment sequencing by mechanism of action

**Figure 4:**
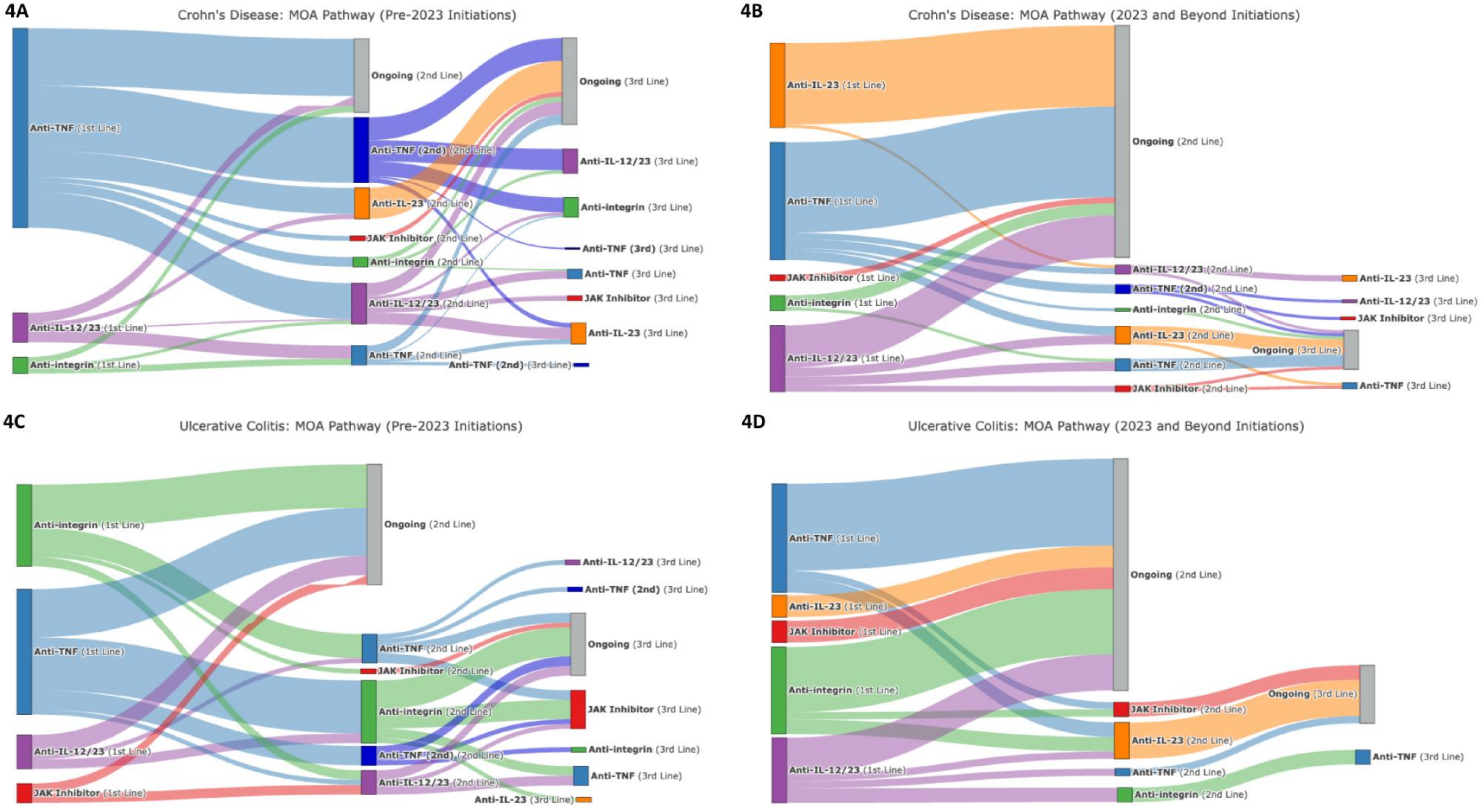
Sequencing of therapies for Crohn’s disease (CD) and ulcerative colitis (UC). The Sankey diagrams illustrate treatment pathways from first- to third-line therapy, stratified by disease and the era of first therapy initiation. (A) CD: MOA pathway (pre-2023 initiations). (B) CD: MOA pathway (2023 and beyond initiations). (C) UC: MOA pathway (pre-2023 initiations). (D) UC: MOA pathway (2023 and beyond initiations).

To visualize the evolution of treatment pathways, Sankey diagrams were generated to illustrate the sequencing of therapies from first- to third-line, stratified by disease and initiation era (Figure 4A-D). In CD, treatment pathways changed substantially over time. For patients initiating therapy pre-2023, the pathway almost exclusively began with an anti-TNF agent (67%). Among those who discontinued this first-line therapy, second-line choices were nearly evenly split between a second anti-TNF (48.1%) and an anti-IL-12/23 agent (42.3%). In contrast, in 2023-2025, first-line therapy was more diverse, with anti-IL-23 agents (30%) used nearly as frequently as anti-TNF agents (41%). For those starting on an anti-TNF and requiring a switch, the subsequent move was overwhelmingly to an anti-IL-23 agent (88.2% of switches). For patients starting on a first-line anti-IL-23 agent, the vast majority remained on this therapy without requiring a switch. In UC, sequencing patterns also evolved. Prior to 2023, first-line therapy was a mix of anti-TNF (41%) and anti-integrin (35%) agents. For patients discontinuing a first-line anti-TNF, the vast majority switched to an anti-integrin agent (88.9%). In 2023-2025, while first-line therapy remained dominated by anti-TNF (41%) and anti-integrin (32%) agents, second-line choices became more varied. Following discontinuation of a first-line anti-TNF agent, patients were switched to anti-IL-23 inhibitors (42.9%) and JAK inhibitors (42.9%) in equal proportions.

### Treatment persistence by mechanism of action

**Figure 5:**
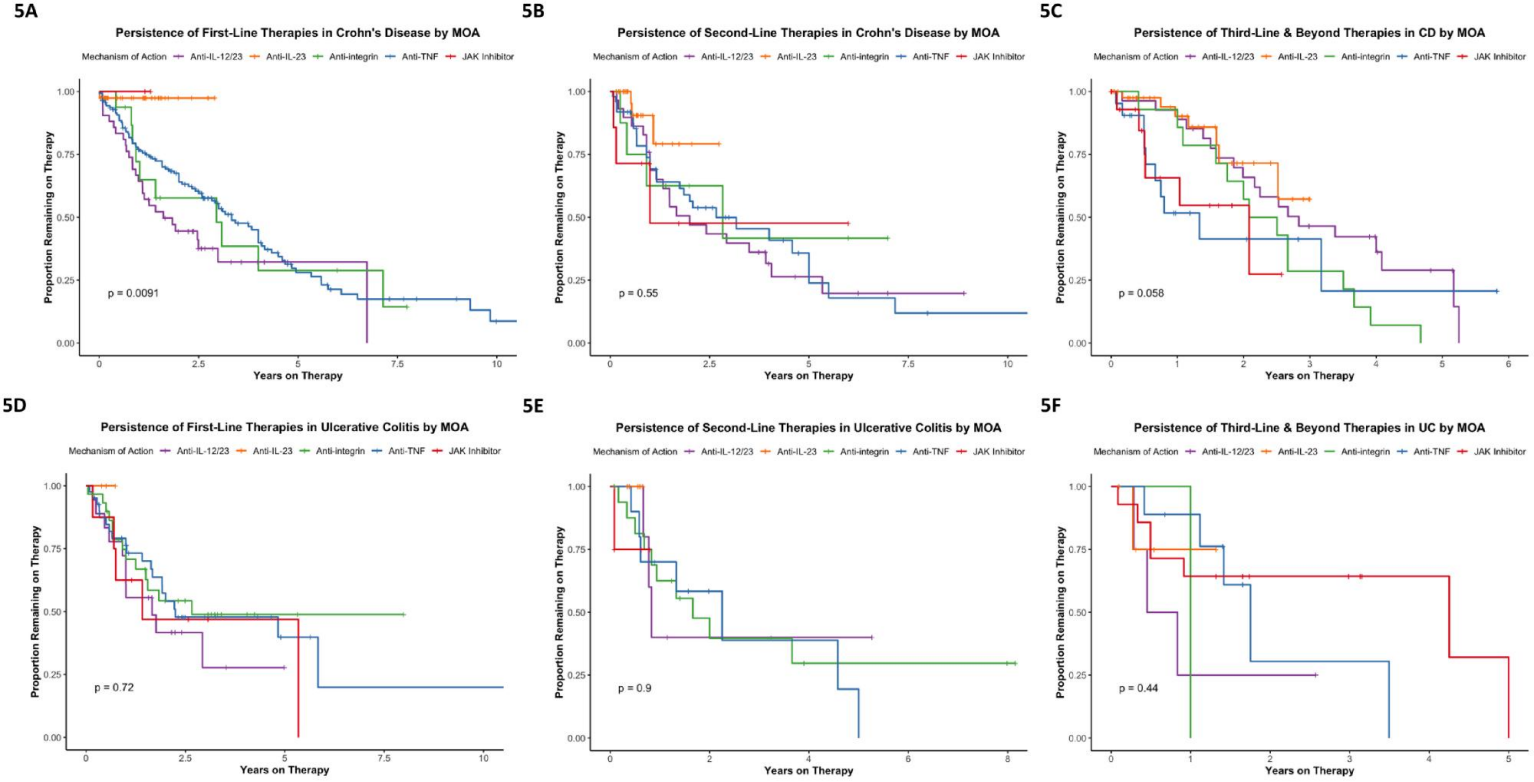
Treatment persistence for Crohn’s disease (CD) and ulcerative colitis (UC) by mechanism of action (MOA). Kaplan-Meier curves show the proportion of patients remaining on therapy over time, stratified by disease and line of therapy. (A) Persistence of first-line therapies in CD by MOA. (B) Persistence of second-line therapies in CD by MOA. (C) Persistence of third-line and beyond therapies in CD by MOA. (D) Persistence of first-line therapies in UC by MOA. (E) Persistence of second-line therapies in UC by MOA. (F) Persistence of third-line & beyond therapies in UC by MOA.

Kaplan-Meier analyses revealed significant differences in treatment persistence based on MOA, particularly in CD (Figure 5A-F). For first-line therapy in CD, pairwise comparisons demonstrated that anti-IL-23 agents had significantly better persistence than all other MOAs: anti-IL-12/23 (*P* = .00026), anti-integrin (*P* = .0062), and anti-TNF (*P* = .011). Anti-TNF agents also showed significantly inferior persistence compared to anti-IL-12/23 agents (*P* = .041). For second-line therapy in CD, no significant differences in persistence were observed among the different MOAs (overall comparison *P* = .55). For third-line and beyond therapies in CD, anti-IL-23 agents demonstrated significantly superior persistence compared to anti-TNF (*P* = .014) and JAK inhibitors (*P* = .033). In UC, treatment persistence was similar across all MOA classes. No statistically significant differences were observed for first-line (log-rank *P* = .72), second-line (log-rank *P* = .90), or third-line and beyond (log-rank *P* = .44) therapies.

### Focused infliximab persistence analysis

To investigate if the effectiveness of infliximab has evolved, we performed a focused analysis of 1-year treatment persistence, grouping new initiations into biennial eras from 2018 onward (Figure S1). The analysis revealed a progressive, stepwise improvement in 1-year persistence with each successive time period. Pairwise comparisons showed that patients initiating infliximab in 2024 and beyond had significantly higher 1-year persistence compared to those who started in 2018–2019 (*P* = .020). This represented a temporal trend just meeting statistical significance (log-rank *P* = .05), demonstrating that patients initiating infliximab in more recent years have a higher probability of remaining on therapy at 1 year compared to those who started in earlier years.

### Predictors of treatment persistence

In univariable analyses of the CD cohort, a penetrating disease phenotype (Montreal B3) was associated with a trend toward poorer treatment persistence compared to inflammatory disease (HR: 1.28, 95% CI: 0.94–1.75, *P* = .11). However, this association did not remain an independent predictor in the multivariable model after adjusting for other factors such as drug class and disease location. Notably, patients with stricturing (B2) and penetrating (B3) disease required a significantly greater number of advanced therapies throughout their disease course compared to those with inflammatory (B1) disease (Kruskal-Wallis *P* < .001) (Figure S3), reflecting the more refractory nature of complicated disease phenotypes. In a multivariable Cox proportional hazards model for the entire advanced therapy-exposed cohort (*n* = 654 therapy episodes), several factors were identified as significant predictors of treatment persistence (Table 2). Compared to anti-TNF therapy, anti-IL-23 agents demonstrated significantly superior persistence (HR: 0.35, 95% CI: 0.19–0.64, *P* = .0008). Current smoking (HR: 1.65, 95% CI: 1.17–2.32, *P* = .0045) and higher line of therapy (HR: 1.14 per line, 95% CI: 1.01–1.30, *P* = .038) were both associated with poorer persistence. Additionally, longer disease duration prior to initiating advanced therapy was associated with better persistence (HR: 0.998 per month, 95% CI: 0.996–1.00, *P* = .037).

**Table 2:** Multivariable Cox proportional hazards models for predictors of advanced therapy discontinuation, by cohort.

Predictor	**All IBD**		**Crohn’s disease**		Ulcerative colitis	
	HR (95% CI)	*P*	HR (95% CI)	*P*	HR (95% CI)	*P*
**Drug class**						
**Anti-IL-12/23 vs. anti-TNF**	1.19 (0.89–1.59)	.239	1.24 (0.88–1.74)	.221	2.18 (1.14–4.19)	.019
**Anti-IL-23 vs. anti-TNF**	0.35 (0.19–0.64)	<.001	0.37 (0.19–0.72)	.004	0.50 (0.07–3.81)	.505
**Anti-integrin vs. anti-TNF**	1.07 (0.77–1.50)	.674	1.24 (0.79–1.93)	.352	1.18 (0.66–2.12)	.579
**JAK inhibitor vs. anti-TNF**	1.09 (0.65–1.81)	.741	1.29 (0.60–2.74)	.515	1.31 (0.62–2.79)	.483
**Subtype: UC vs. CD**	1.15 (0.87–1.52)	.316	—	—	—	—
**Line of therapy (per line)**	1.14 (1.01–1.30)	.038	1.04 (0.89–1.22)	.619	1.19 (0.95–1.49)	.131
**Age at diagnosis (per year)**	0.99 (0.98–1.00)	.193	1.00 (0.98–1.01)	.746	0.99 (0.96–1.02)	.442
**Smoking history**						
**Current smoker vs. nonsmoker**	1.65 (1.17–2.32)	.004	1.59 (1.09–2.32)	.016	2.11 (0.70–6.39)	.185
**Ex-smoker vs. nonsmoker**	1.42 (0.82–2.48)	.214	1.60 (0.90–2.83)	.107	—a	—a
**Disease duration at initiation (per month)**	0.998 (0.996–1.000)	.037	0.999 (0.997–1.002)	.535	0.994 (0.989–0.999)	.013
**Montreal disease behavior**						
**B2 (stricturing) vs. B1 (inflammatory)**	—	—	0.84 (0.60–1.17)	.299	—	—
**B3 (penetrating) vs. B1 (inflammatory)**	—	—	1.13 (0.81–1.59)	.470	—	—
**Disease location**						
**Colonic (L2) vs. ileo-colonic (L3)**	—	—	0.88 (0.53–1.44)	.599	—	—
**Ileal (L1) vs. ileo-colonic (L3)**	—	—	0.54 (0.37–0.80)	.002	—	—
**Perianal disease (yes vs. no)**	—	—	1.05 (0.80–1.39)	.728	—	—
**UC extent**						
**Left-sided colitis vs. proctitis**	—	—	—	—	2.19 (0.50–9.58)	.298
**Pancolitis vs. proctitis**	—	—	—	—	1.31 (0.32–5.44)	.708

In a sub-analysis restricted to patients with CD (*n* = 481 therapy episodes), the superior persistence of anti-IL-23 therapy remained significant (HR: 0.37, 95% CI: 0.19–0.72, *P* = .0035). Current smoking was also associated with poorer persistence in CD patients (HR: 1.59, 95% CI: 1.09–2.32, *P* = .016), while ileal disease location (Montreal L1), compared to ileo-colonic (L3), was associated with better persistence (HR: 0.54, 95% CI: 0.37–0.80, *P* = .0019). For patients with UC (*n* = 173 therapy episodes), longer disease duration prior to initiating advanced therapy was associated with better persistence (HR: 0.994 per month, 95% CI: 0.989–0.999, *P* = .013), suggesting that these patients likely had a milder disease course before requiring escalation. Interestingly, anti-IL-12/23 agents demonstrated poorer persistence compared to anti-TNF in UC (HR: 2.18, 95% CI: 1.14–4.19, *P* = .019). Each subsequent line of therapy showed a trend toward poorer persistence, though this did not reach statistical significance (HR: 1.19 per line, 95% CI: 0.95–1.49, *P* = .13).

### Sensitivity analyses to address confounding by indication

Baseline phenotype distributions stratified by first-line MOA are presented in Table S1 to allow direct inspection of channeling between treatment classes. To further address the possibility that anti-IL-23 might be preferentially channeled to patients with milder disease, 3 additional analyses were performed. First, in a sensitivity multivariable Cox model restricted to patients with complicated CD (Montreal stricturing [B2] or penetrating [B3] phenotype; *n* = 224 therapy episodes from 108 unique patients; 124 discontinuation events), the anti-IL-23 versus anti-TNF persistence advantage was preserved (adjusted HR 0.34, 95% CI 0.12 to 0.93, *P* = .035; concordance 0.644; Table S2). Second, an inverse-probability-of-treatment-weighted Cox analysis comparing anti-IL-23 versus anti-TNF first-line in CD (restricted to initiations from January 2020 onward to provide reasonable temporal overlap; *n* = 143 episodes, 56 events) was attempted; covariate overlap between groups was insufficient for stable estimation (effective sample size in the anti-IL-23 arm collapsed from 38 to 5.5 after weighting and the doubly-robust covariate-adjusted model was rank-deficient), and the weighted estimate is reported as a methodological diagnostic only (Table S3 and Figure S4). Third, *E*-values were computed to bound the strength of unmeasured confounding required to explain away the observed anti-IL-23 advantage. For the headline All-IBD multivariable Cox HR (0.35; 95% CI 0.19 to 0.64), the *E*-value was 3.52 (CI limit 2.06); comparable values were obtained for the CD-only multivariable model (*E*-value 3.37; CI limit 1.82) and the B2/B3-restricted sensitivity model (*E*-value 3.60; CI limit 1.29). An unmeasured confounder would therefore need to be associated with both anti-IL-23 use and treatment persistence by a risk ratio of at least 3.5, conditional on measured covariates, to fully explain away the observed advantage.

### Surgical outcomes

Analysis of early surgical outcomes was performed on 504 patients (330 CD, 174 UC) with follow-up censored at 35 months from diagnosis. There was no statistically significant difference in 35-month surgery-free survival for patients diagnosed before 2023 versus 2023-and-beyond for either CD (15/229 [6.6%] vs. 4/101 [4.0%], *P* = .9) or UC (3/122 [2.5%] vs. 1/52 [1.9%], *P* = .9) (Figure S2A). In a multivariable logistic regression model, independent predictors of requiring surgery included younger age at diagnosis (OR: 0.94 per year, 95% CI: 0.91–0.97, *P* < .001), a diagnosis of CD versus UC (OR for UC: 0.34, 95% CI: 0.13–0.74, *P* = .012), and current smoking (OR for nonsmokers: 0.33, 95% CI: 0.16–0.74, *P* = .005). The number of preoperative advanced therapies was not an independent predictor (OR: 0.76, 95% CI: 0.53–1.03, *P* = .10). Among patients with CD, Montreal disease behavior at diagnosis was a strong predictor of early surgery, with significantly higher risk for those with stricturing (B2) or penetrating (B3) disease compared to inflammatory (B1) disease (log-rank *P* < .001) (Figure S2B).

## Discussion

These findings should be interpreted in the context of a single-center tertiary cohort and are likely to be influenced by referral patterns, confounding by indication, and prescriber preference. In the context of a rapidly rising IBD burden in the Middle East, our study extends a small but growing body of real-world IBD therapeutics literature from the region. A recent Saudi retrospective study reported patterns of biologic switching in IBD patients between 2017 and 2022, while a narrative review from the same region synthesized 7 studies of biologic utilization, both highlighting anti-TNF agents as the predominant first-line choice and identifying unmet needs in longitudinal outcome data.^13^^,^^14^ Our cohort differs in several important respects: it captures a more recent time horizon extending into 2025, spans the transition into the multi-mechanism era with newer anti-IL-23 and JAK inhibitor agents, and links prescribing patterns directly to treatment persistence and early surgical outcomes for both CD and UC. Our analysis reveals two key findings: first, a dramatic evolution in prescribing patterns for CD since the beginning of 2023, moving away from anti-TNF dominance toward newer mechanisms of action; and second, that the relevance of this shift in clinical practice is supported by our real-world data showing superior treatment persistence of some of these newer agents, particularly those targeting the anti-interleukin pathway in specific settings. This evolution is consistent with the increasing complexity of IBD in the region, reflecting a move toward more individualized care.^15^

The approach to therapy initiation differed between CD and UC, yet both demonstrated a significant trend toward earlier intervention in recent years. For UC, while a “step-up” approach remains standard for mild-to-moderate disease,^16^ resulting in a persistently high proportion of therapy-naive patients, our data reveals a significant acceleration in the use of advanced therapies for those who require them. The median time from diagnosis to first advanced therapy in UC patients dropped dramatically from 37.0 months pre-2023 to just 3.0 months from 2023 onward. Consequently, a large proportion of UC patients are appropriately managed without ever needing advanced therapies, explaining the stable, high percentage of therapy-naive patients observed in our cohort. This suggests that for UC patients needing escalation, decisions are increasingly driven by early risk stratification (eg severe or extensive disease) rather than a prolonged “wait and see” period.

Similarly, the management of moderate-to-severe CD saw a pronounced shift toward earlier intervention. A key finding of our study is the significant reduction in the time from diagnosis to the initiation of first advanced therapy, dropping from a median of 23.0 months before 2023 to just 2.0 months from 2023 onward. This trend aligns with a global shift away from the traditional “step-up” approach toward earlier intervention in patients with moderate-to-severe disease or risk factors for poor outcomes.^2^ Seminal studies such as CALM, REACT, and PROFILE have provided strong evidence that a “top-down” or early aggressive treatment strategy in CD can alter the natural history of the disease and improve long-term outcomes, including reducing rates of surgery and hospitalization.^17–19^ Our observation that a greater proportion of newly diagnosed CD patients are starting advanced therapies within the first year is a strong indicator that this evidence is being translated into clinical practice. This finding does not suggest a delayed response to aggressive disease phenotypes, but rather the earlier adoption of risk stratification at diagnosis, a cornerstone of modern IBD management. By identifying and treating high-risk patients earlier, the goal is to prevent the progression to complicated, disabling disease.

The displacement of anti-TNF agents from their long-held position as the default first-line therapy for CD is striking and reflects a global trend, but appears particularly accelerated in our cohort. First-line anti-TNF use in CD declined from 76% to 42%, with anti-IL-23 agents now accounting for 32% of first-line initiations. This is likely facilitated by a practice environment without the payer- or regulator-mandated positioning restrictions seen elsewhere, allowing for quicker adoption of new evidence.^20–22^ Our treatment sequencing analysis confirms this, showing that from 2023 onward, clinicians are not only choosing anti-IL-23 agents first-line but are also overwhelmingly selecting them as the preferred second-line therapy following anti-TNF failure.^23^^,^^24^ This rapid evolution in clinical decision-making provides a clear, real-world snapshot of a treatment landscape in transformation.^6^^,^^25^

Our persistence data provide observational evidence consistent with the prescribing shift in CD. The findings from the first-line Kaplan-Meier analysis demonstrated statistically significant superiority of anti-IL-23 agents over all comparator classes, including anti-TNF. The multivariate Cox model consistently demonstrated that anti-IL-23 agents are associated with a significantly lower risk of treatment discontinuation in CD compared to anti-TNF therapy. Furthermore, anti-IL-23 agents maintained significantly superior persistence even in the third-line and beyond setting, providing additional support for their role across the treatment algorithm. This superior persistence, a proxy for long-term effectiveness and tolerability, is consistent with the shift in prescribing habits.^26^ Furthermore, our models confirmed established risk factors for treatment failure, such as current smoking and later lines of therapy, while also highlighting that in CD, an ileal disease location was associated with better drug survival.

A notable finding in the updated analysis is the emergence of statistically significant shifts in UC second-line prescribing patterns (*P* = .048), suggesting that even for UC, clinicians are increasingly diversifying their therapeutic approach in the second-line setting, with greater use of anti-IL-23 and JAK inhibitors. However, the stability of first-line prescribing patterns and the lack of significant differences in persistence among MOAs in UC suggest that the treatment algorithm for this condition is more established, with anti-TNF and anti-integrin agents remaining effective cornerstone therapies.^27^ Interestingly, longer disease duration prior to starting advanced therapy in UC was protective against discontinuation, perhaps reflecting a cohort with historically milder disease requiring later escalation.

The accelerated shift observed from 2023 onward reflects a confluence of regulatory, institutional, and guideline-level factors. A dedicated IBD specialist has been embedded within the center’s gastroenterology service since 2021, and the program gained designation as a flagship IBD service in 2024. By the end of the study period, the service comprised 3 dedicated IBD physicians, an IBD specialist nurse, and a dedicated multidisciplinary team comprising IBD dietitian, clinical psychologist, clinical pharmacist, and IBD surgeons, supported by a weekly multidisciplinary meeting and routine proactive therapeutic drug monitoring for anti-TNF agents. Formal adoption of treat-to-target principles in line with international (STRIDE-II) and regional (UAE consensus) recommendations, together with a strategy of early effective advanced therapy, coincided with the regulatory approval and clinical availability of selective anti-IL-23 inhibitors and JAK-1 inhibitors within a narrow window during 2022–2023. We are unable to fully disentangle the relative contribution of each factor in this observational design, and acknowledge this as a limitation.

A particularly noteworthy finding is the progressive improvement in 1-year infliximab persistence over successive biennial eras, with the most recent cohort (2024 and beyond) showing significantly better persistence than the earliest cohort (2018–2019). This suggests that even as newer agents become preferred, the effectiveness of established therapies can be optimized. This improvement is likely multifactorial, reflecting better patient selection, the routine use of proactive therapeutic drug monitoring to guide dosing, greater co-prescription with immunomodulators, and the introduction of subcutaneous formulations.^28–33^ This highlights that established therapies are not obsolete but can be used more effectively within a modern, optimized treatment strategy.

In our cohort, longer disease duration prior to first advanced therapy was associated with better persistence in the multivariable Cox model. This appears, at first glance, opposite in direction to the PROFILE trial, in which an early aggressive “top-down” strategy from diagnosis was superior to delayed accelerated step-up management for newly diagnosed CD, both at the 48-week primary endpoint and at the recently reported 4-year follow-up.^19^^,^^34^ The two findings are not contradictory; rather, they reflect different study designs and case-mixes. PROFILE was a within-trial randomized comparison among newly diagnosed patients (median 12 days from diagnosis to enrolment) testing the effect of treatment strategy at the start of the disease course, and the long-term follow-up showed that early effective therapy was associated with reduced disease progression, hospitalization and surgery over 4 years. Our cohort is observational and cross-decade, including patients who reached first advanced therapy at any point during a real-world disease course of variable duration. In this setting, patients with longer disease duration before reaching first advanced therapy represent a “survivor” subgroup who tolerated conventional therapy for years and likely have inherently milder, less aggressive disease, accounting for the direction of effect we observe in the multivariable model. This interpretation is particularly evident in our UC subgroup, where historical “step-up” management of milder disease is well established.

Regarding surgical outcomes, our study reinforces the critical role of disease phenotype in predicting prognosis. A complicated Montreal B2 or B3 phenotype at diagnosis remains the most potent predictor of needing early surgery, and these patients also cycle through more advanced therapies. This underscores the necessity of early risk stratification to identify patients who may benefit from aggressive, top-down therapeutic approaches from diagnosis.^35^ Interestingly, despite the significant shifts in medical therapy, we did not observe a change in the rate of first major surgery within 35 months of diagnosis between the two eras. The overall low rate of IBD-related abdominal surgery in our cohort likely reflects both a high degree of patient and family apprehensiveness toward abdominal surgery in this population, which is well recognized in clinical practice across the region, and the early access to a broad portfolio of advanced therapies that may delay or avert the need for surgical intervention. Nevertheless, the lack of a significant between-era difference suggests that the impact of newer therapies on altering the natural history of the most severe disease may require longer follow-up to become apparent, or that a subset of patients with aggressive disease will inevitably require early surgical intervention. The multivariable analysis identified younger age, CD diagnosis, and current smoking as significant predictors of surgery. The use of combination advanced therapy highlights a small subset of patients with highly refractory disease, predominantly CD (7/9 patients), who are not adequately controlled by sequential monotherapy and represent a key area of unmet need.

To our knowledge, this is among the first real-world evaluations from the Middle East to comprehensively document the evolution of advanced therapy utilization and persistence, capturing the paradigm shift from the anti-TNF-dominant period into the modern, multi-MOA era. Furthermore, the study’s setting within a large, tertiary referral center and its focus on a homogenous Arab population, of whom 80% were Emirati, provides crucial insights for a group that is underrepresented in IBD literature. Our prospectively maintained registry also provides granular, longitudinal data on therapy use. However, we acknowledge several limitations, many of which are consistent with those described in the first part of this study. Our findings may not be generalizable to all practice settings, as tertiary centers are susceptible to referral bias from more complex cases. It is also important to state that while we have used persistence as a key marker for assessment, this is not the same as treatment effectiveness. The detailed assessment of treatment effectiveness, incorporating clinical and biomarker outcomes, will form part of separate real-world evidence assessments for individual therapies and mechanisms of action in future publications.^36^ In addition, contemporaneous endoscopic disease activity scores (eg SES-CD) and fecal calprotectin levels at the time of each line initiation were not consistently captured across the full 2015–2025 study period; residual confounding by indication therefore cannot be fully excluded. We attempted to bound this concern through a sensitivity multivariable Cox model restricted to complicated (Montreal B2/B3) CD and through E-value analysis (Results; Table S2), both of which were consistent with our headline findings. Furthermore, our analysis does not capture the specific reasons for treatment discontinuation (eg, primary non-response, loss of response, adverse events). Finally, the follow-up time for the most recently introduced therapies and for the 2023 and beyond era is necessarily short, limiting our ability to assess long-term outcomes and the true impact on altering the natural history of the disease.

## Conclusion

In conclusion, this study of our IBD cohort shows that during 2015–2025, the IBD treatment landscape in our region has transformed in response to a growing disease burden and an expanding therapeutic toolkit. For CD, there has been a clear and rapid shift toward newer, more persistent therapeutic classes, initiated significantly earlier in the disease course. For UC, advanced therapies are also being initiated markedly earlier for patients requiring escalation, and significant shifts in second-line prescribing have emerged. While infliximab persistence has improved, a complicated disease phenotype remains a key driver for early surgery. As the burden of IBD continues to grow in the region, longer-term follow-up of this cohort will be essential to determine whether this evolution in medical management will ultimately translate into a reduction in the long-term burden of surgery and disability in IBD.

## Supplementary Material

otag057_Supplementary_Data

## Data Availability

The data that support the findings of this study are available from the corresponding author upon reasonable request. The data are not publicly available due to their sensitive nature, containing information that could compromise patient confidentiality and privacy.
