# Supplementary material for: Paradigm shift in inflammatory bowel disease management—advanced therapy utilization, persistence, and outcomes in a United Arab Emirates cohort during 2015-2025
Source: Crohns Colitis 360. 2026 Jun 16;8(3):otag057. doi: 10.1093/crocol/otag057 (PMC13345672; doi:10.1093/crocol/otag057)

**Supplemental Material**

*Paradigm Shift in Inflammatory Bowel Disease Management: Advanced Therapy Utilisation, Persistence, and Outcomes in a United Arab Emirates Cohort during 2015–2025*

**Supplementary Table S1. Baseline phenotype distribution stratified by first-line mechanism of action (advanced-therapy-exposed cohort, n = 381).**

***Crohn's disease (n = 275 first-line initiations)***

| **Phenotype variable** | **Anti-TNF (n=177)** | **Anti-integrin (n=16)** | **Anti-IL-12/23 (n=42)** | **Anti-IL-23 (n=38)** | **JAK inhibitor (n=2)** |
| --- | --- | --- | --- | --- | --- |
| Montreal disease behaviour |  |  |  |  |  |
| B1 (inflammatory) | 89 (50.3%) | 12 (75.0%) | 28 (66.7%) | 31 (81.6%) | 2 (100.0%) |
| B2 (stricturing) | 48 (27.1%) | 4 (25.0%) | 8 (19.0%) | 5 (13.2%) | 0 (0.0%) |
| B3 (penetrating) | 40 (22.6%) | 0 (0.0%) | 6 (14.3%) | 2 (5.3%) | 0 (0.0%) |
| Disease location |  |  |  |  |  |
| L1 (ileal) | 33 (18.6%) | 5 (31.2%) | 14 (33.3%) | 16 (42.1%) | 1 (50.0%) |
| L2 (colonic) | 19 (10.7%) | 3 (18.8%) | 2 (4.8%) | 3 (7.9%) | 0 (0.0%) |
| L3 (ileo-colonic) | 125 (70.6%) | 8 (50.0%) | 26 (61.9%) | 19 (50.0%) | 1 (50.0%) |
| Perianal disease (yes) | 79 (44.6%) | 2 (12.5%) | 11 (26.2%) | 3 (7.9%) | 0 (0.0%) |
| Prior IBD-related surgery (yes) | 39 (22.0%) | 2 (12.5%) | 3 (7.1%) | 2 (5.3%) | 0 (0.0%) |

***Ulcerative colitis (n = 106 first-line initiations)***

| **Phenotype variable** | **Anti-TNF (n=47)** | **Anti-integrin (n=30)** | **Anti-IL-12/23 (n=18)** | **Anti-IL-23 (n=3)** | **JAK inhibitor (n=8)** |
| --- | --- | --- | --- | --- | --- |
| UC extent |  |  |  |  |  |
| E1 (proctitis) | 1 (2.1%) | 1 (3.3%) | 2 (11.1%) | 0 (0.0%) | 1 (12.5%) |
| E2 (left-sided colitis) | 14 (29.8%) | 5 (16.7%) | 4 (22.2%) | 2 (66.7%) | 1 (12.5%) |
| E3 (pancolitis) | 32 (68.1%) | 24 (80.0%) | 12 (66.7%) | 1 (33.3%) | 6 (75.0%) |
| Prior IBD-related surgery (yes) | 3 (6.4%) | 4 (13.3%) | 0 (0.0%) | 0 (0.0%) | 0 (0.0%) |

*Supplementary Table S1 added in revision (Reviewer 3, R3-R-2) to allow the reader to assess channelling of treatment selection by baseline phenotype. Percentages are within the column (first-line MOA group). UC extent categories Proctitis, Left-sided colitis and Pancolitis correspond to Montreal E1, E2 and E3 respectively. The first-line denominators (CD = 275 episodes; UC = 106 episodes) sum to 381 patients exposed to at least one advanced therapy.*

**Supplementary Table S2. Sensitivity multivariable Cox proportional hazards model for predictors of advanced therapy discontinuation, restricted to patients with complicated (Montreal B2 stricturing or B3 penetrating) Crohn's disease.**

| **Predictor** | **Reference category** | **Adjusted HR (95% CI)** | **p value** |
| --- | --- | --- | --- |
| Drug class |  |  |  |
| Anti-IL-12/23 | Anti-TNF | 1.11 (0.67 – 1.86) | 0.685 |
| Anti-IL-23 | Anti-TNF | 0.34 (0.12 – 0.93) | 0.035 |
| Anti-integrin | Anti-TNF | 1.43 (0.76 – 2.70) | 0.273 |
| JAK inhibitor | Anti-TNF | 3.60 (1.26 – 10.29) | 0.017 |
| Line of therapy (per line) | 1st line | 0.99 (0.80 – 1.23) | 0.931 |
| Age at diagnosis (per year) | — | 1.01 (0.99 – 1.02) | 0.583 |
| Smoking history |  |  |  |
| Current smoker | Non-smoker | 1.53 (0.96 – 2.44) | 0.074 |
| Ex-smoker | Non-smoker | 0.93 (0.41 – 2.13) | 0.863 |
| Disease duration at initiation (per month) | — | 1.00 (1.00 – 1.00) | 0.693 |
| Montreal behaviour |  |  |  |
| B3 (penetrating) | B2 (stricturing) | 1.47 (1.00 – 2.16) | 0.050 |
| Disease location |  |  |  |
| Colonic (L2) | Ileo-colonic (L3) | 1.79 (0.82 – 3.92) | 0.142 |
| Ileal (L1) | Ileo-colonic (L3) | 0.62 (0.36 – 1.06) | 0.081 |
| Perianal disease (yes) | No | 0.89 (0.60 – 1.30) | 0.545 |

*n = 224 therapy episodes from 108 unique patients; 124 discontinuation events. Concordance index 0.644; likelihood-ratio test χ² = 29.96, df = 13, p = 0.005. The B1 (inflammatory) phenotype was excluded by design (this analysis tests the persistence advantage in the most severe phenotype subgroup, where channelling away from anti-IL-23 would most plausibly bias the comparison). HR, hazard ratio; CI, confidence interval. Reference categories shown in the second column.*

**Supplementary Table S3. Inverse-probability-of-treatment-weighted (IPTW) propensity-score analysis comparing anti-IL-23 with anti-TNF first-line use in Crohn's disease, restricted to initiations from January 2020 onwards.**

*Panel A. Standardised mean differences before and after IPTW.*

| **Covariate** | **Type** | **Unadjusted SMD** | **Weighted SMD** | **Threshold (\|SMD\| < 0.1)** |
| --- | --- | --- | --- | --- |
| Propensity score | Distance | 1.44 | −0.17 | — |
| Age at diagnosis | Continuous | 0.71 | −0.19 | Not balanced |
| Smoking history (non-smoker) | Binary | −0.33 | −0.03 | Balanced |
| Smoking history (current smoker) | Binary | 0.32 | −0.03 | Balanced |
| Smoking history (ex-smoker) | Binary | 0.07 | 0.10 | Not balanced |
| Disease duration at initiation (months) | Continuous | 0.28 | 0.05 | Balanced |
| Montreal B1 (inflammatory) | Binary | 0.65 | −0.28 | Not balanced |
| Montreal B2 (stricturing) | Binary | −0.30 | 0.57 | Not balanced |
| Montreal B3 (penetrating) | Binary | −0.52 | −0.29 | Not balanced |
| Location: ileo-colonic (L3) | Binary | −0.32 | 0.33 | Not balanced |
| Location: colonic (L2) | Binary | −0.23 | −0.23 | Not balanced |
| Location: ileal (L1) | Binary | 0.52 | −0.20 | Not balanced |
| Perianal disease (yes) | Binary | −0.88 | 0.45 | Not balanced |

*Panel B. IPTW Cox proportional hazards model for advanced therapy discontinuation: anti-IL-23 versus anti-TNF.*

| **Treatment comparison** | **HR (95% CI)** | **Wald p value** |
| --- | --- | --- |
| Anti-IL-23 versus anti-TNF (IPTW unadjusted) | 0.02 (0.002 – 0.24) | 0.002 |
| Anti-IL-23 versus anti-TNF (IPTW + covariate-adjusted, doubly robust) | Not estimable (rank-deficient) | — |

*Cohort: 143 first-line CD therapy episodes initiated from 1 January 2020 onwards (anti-TNF n = 105; anti-IL-23 n = 38); 56 discontinuation events. Propensity scores estimated by logistic regression on age at diagnosis, smoking history, disease duration at initiation, Montreal behaviour, disease location and perianal disease, with average treatment effect (ATE) weighting. The maximum stabilised weight in the anti-IL-23 arm was 71.99; the effective sample size of the anti-IL-23 arm collapsed from 38 to 5.5 after weighting. Nine of 12 baseline covariates remained imbalanced (|SMD| > 0.1) after weighting (Panel A), and the doubly-robust IPTW + covariate-adjusted Cox model was rank-deficient (variance matrix singular) and could not be estimated (Panel B). The IPTW point estimate is therefore reported as an exploratory diagnostic only; the B2/B3-restricted sensitivity Cox model (Supplementary Table S2) provides the more reliable assessment of confounding by indication. SMD, standardised mean difference; HR, hazard ratio; CI, confidence interval; ATE, average treatment effect; IPTW, inverse-probability-of-treatment-weighted.*

**Supplementary Figure S1. Evolution of 1-year infliximab persistence by biennial era of initiation.**

Kaplan–Meier analysis of 1-year persistence on infliximab, stratified by biennial era of treatment initiation (2018–2019, 2020–2021, 2022–2023, 2024 and beyond). A statistically significant improvement in 1-year persistence was observed across successive biennia (overall log-rank p = 0.05); pairwise comparison demonstrated significantly better persistence in the 2024-and-beyond cohort compared with the 2018–2019 cohort (p = 0.020).


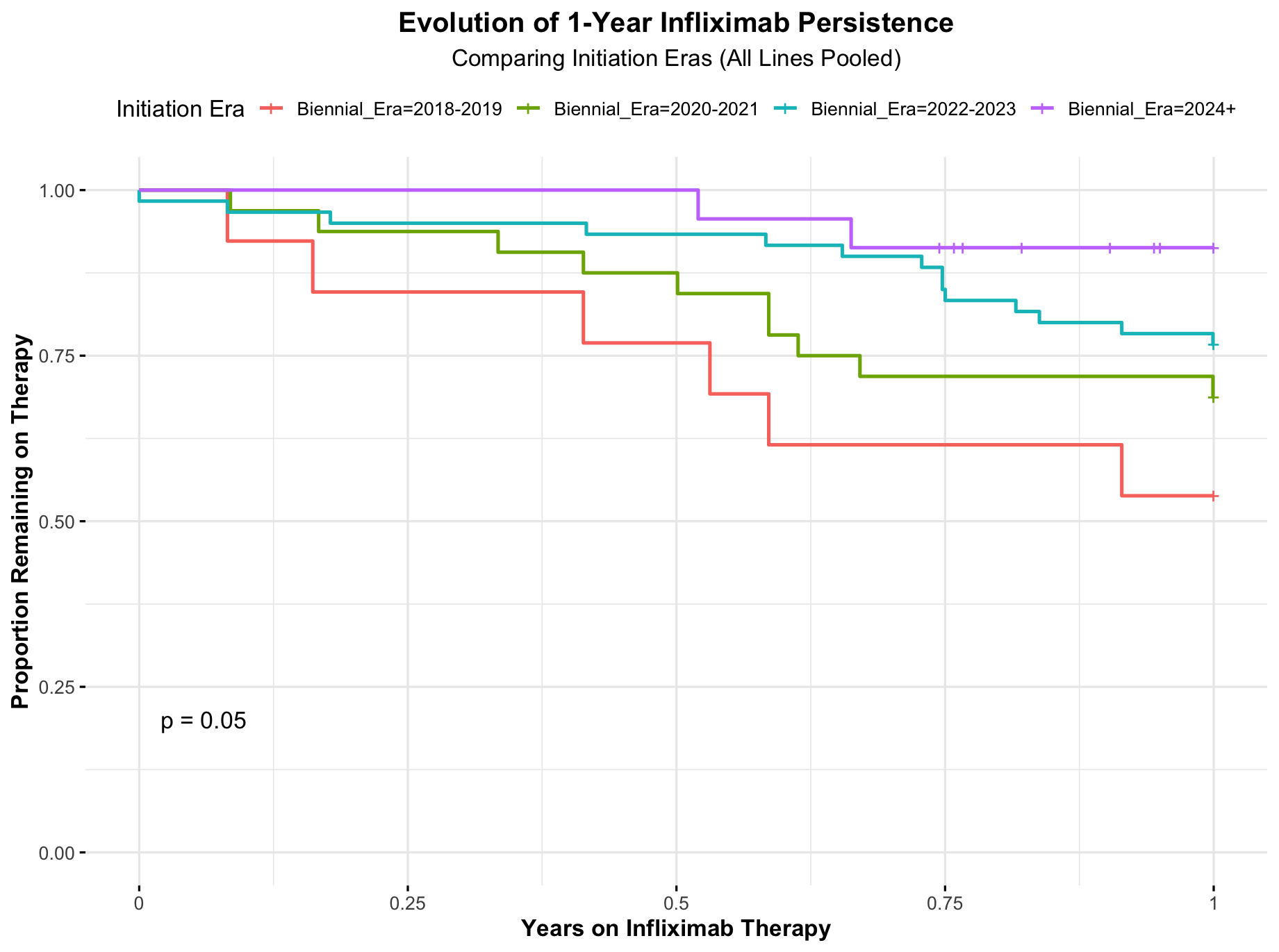


**Supplementary Figure S2. 35-month surgery-free survival.**

(A) Kaplan–Meier curves comparing 35-month surgery-free survival between the pre-2023 and 2023-and-beyond eras. No significant difference was observed for either Crohn's disease (CD; p = 0.9) or ulcerative colitis (UC; p = 0.9). Total surgical events: 23 (CD: 15/232 [6.5%] pre-2023 versus 4/102 [3.9%] in 2023+; UC: 3/122 [2.5%] pre-2023 versus 1/53 [1.9%] in 2023+). With this small event count, the analysis is underpowered and findings should be interpreted as exploratory. (B) In CD, 35-month surgery-free survival was significantly worse for patients with a complicated Montreal phenotype (B2 stricturing or B3 penetrating) at diagnosis compared with those with inflammatory (B1) disease (log-rank p < 0.001).


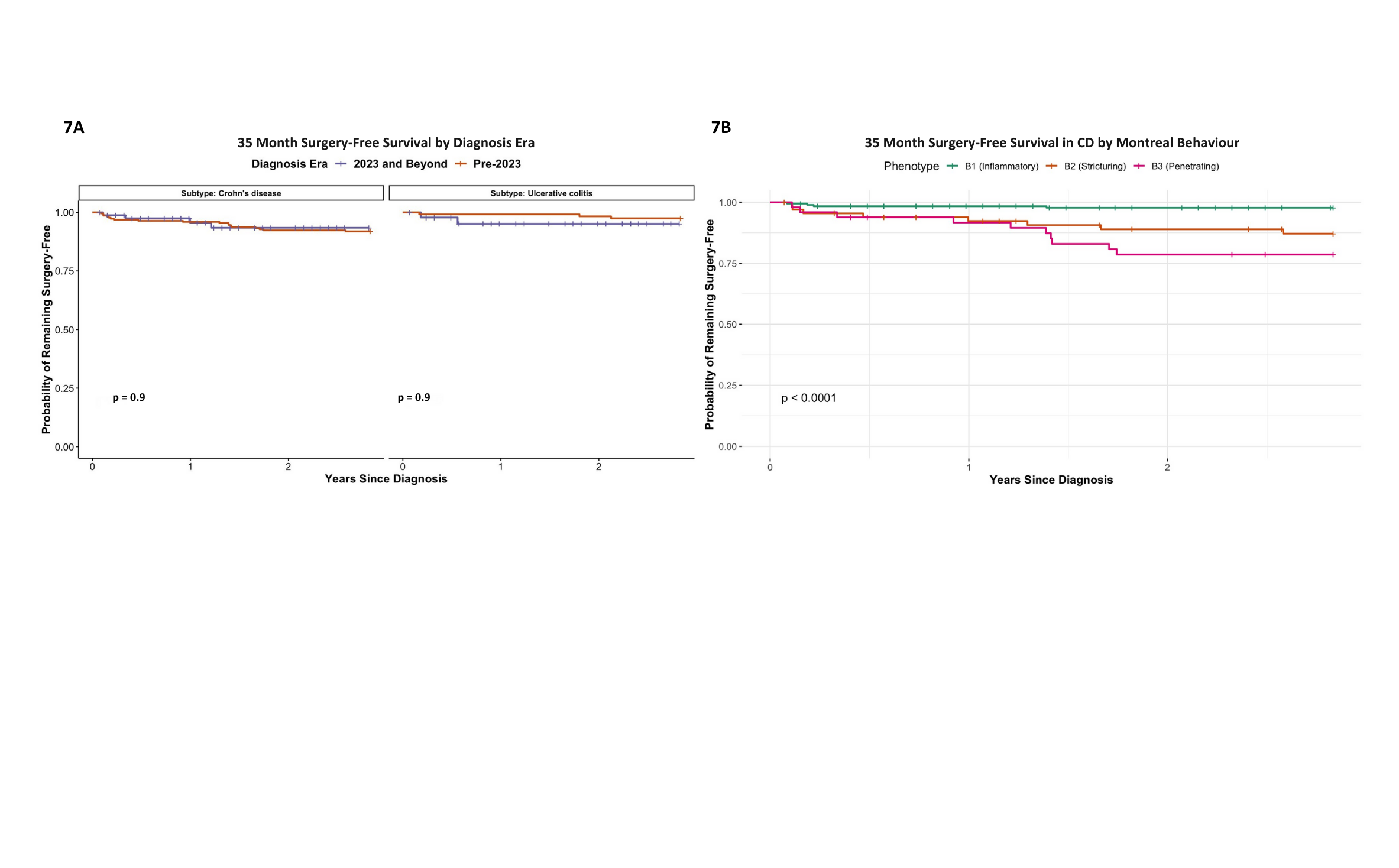


**Supplementary Figure S3. Total advanced therapy exposure in Crohn's disease, stratified by Montreal disease behaviour at diagnosis.**

Violin plot showing the total number of advanced therapy episodes received by patients with Crohn's disease, stratified by Montreal disease behaviour at diagnosis (B1 inflammatory, B2 stricturing, B3 penetrating). Patients with stricturing (B2) and penetrating (B3) disease required a significantly greater number of advanced therapies than those with inflammatory (B1) disease (Kruskal–Wallis χ² = 28.47, df = 2, p = 6.6 × 10⁻⁷).


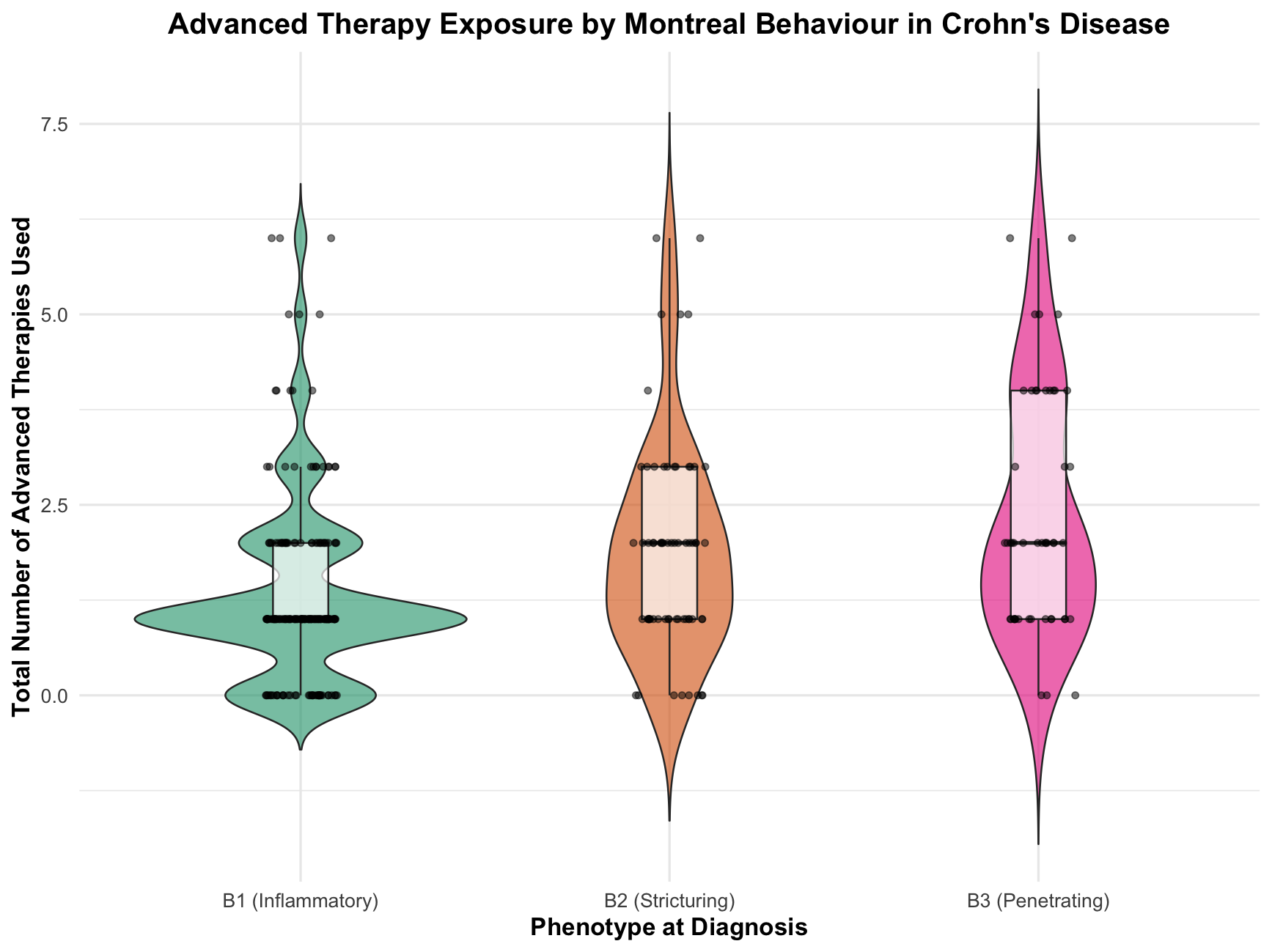


**Supplementary Figure S4. Propensity-score overlap density plot for the inverse-probability-of-treatment-weighted analysis of anti-IL-23 versus anti-TNF first-line use in Crohn's disease (initiations from January 2020 onwards).**

Density plots of the estimated propensity score for anti-IL-23 treatment, before (left panel) and after (right panel) inverse-probability-of-treatment weighting. Marked non-overlap between the anti-IL-23 (treated) and anti-TNF (control) groups is evident before weighting; weighting reduces but does not resolve the non-overlap, with the effective sample size of the anti-IL-23 arm collapsing from 38 to 5.5 patients after weighting and nine of 12 baseline covariates remaining imbalanced (Supplementary Table S3). These diagnostics support the conclusion that channelling between anti-IL-23 and anti-TNF first-line CD initiations in this cohort is too extreme for stable propensity-score-based estimation, and that the B2/B3-restricted sensitivity Cox model (Supplementary Table S2) provides the more reliable assessment of confounding by indication.


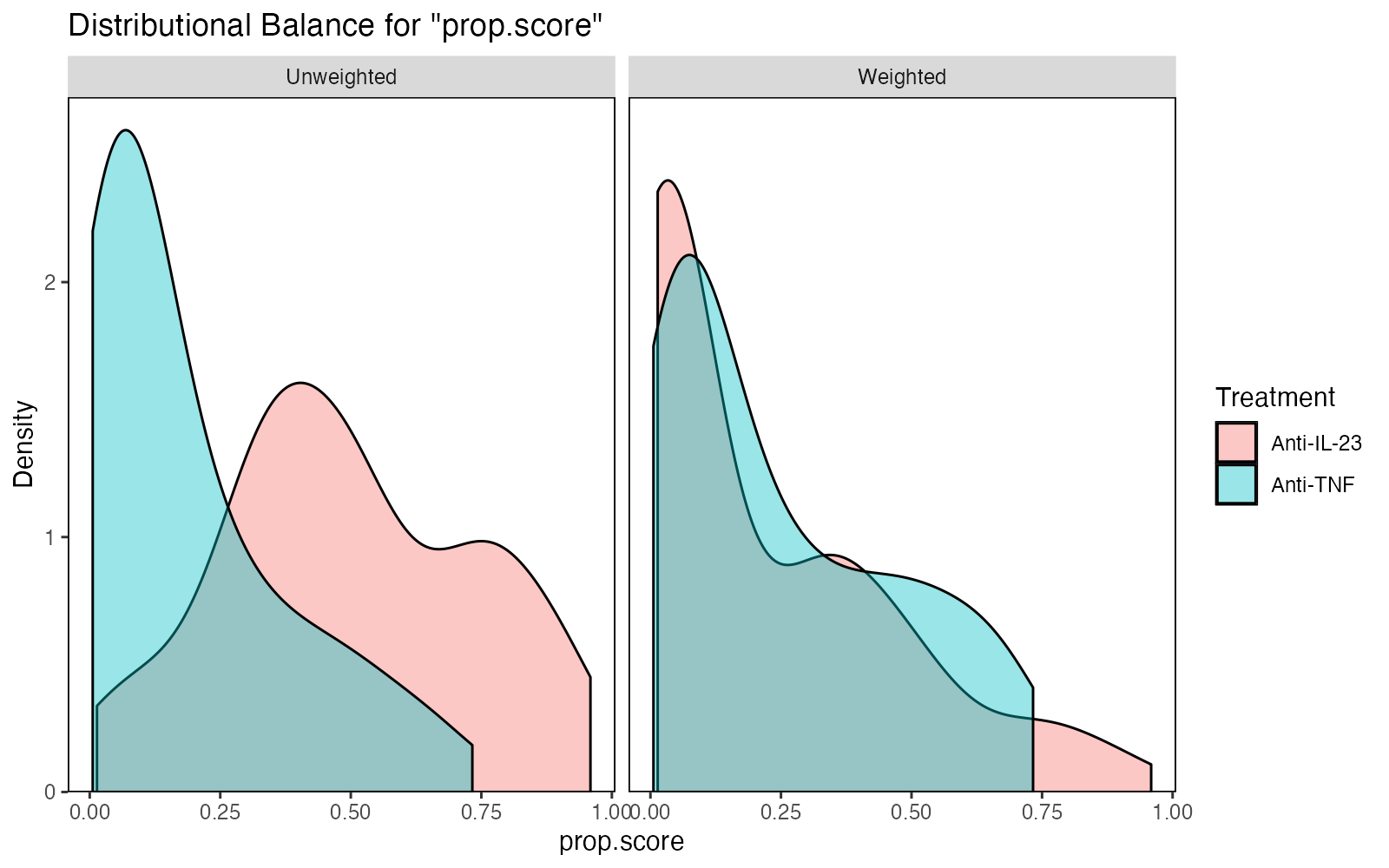

Supplement: otag057_Supplementary_Data [file otag057_supplementary_data.docx]
